# Vraie macrodactylie avec orientation nerveuse

**DOI:** 10.11604/pamj.2018.31.61.15303

**Published:** 2018-09-27

**Authors:** Siham Nasri, Asmae Oulad Amer, Narjisse Aichouni, Imane Kamaoui, Imane Skiker

**Affiliations:** 1 Service de Radiologie, CHU Mohammed VII, 20100 Oujda, Maroc

**Keywords:** Macrodactylie, doigt, malformation, nerf, Macrodactylia, finger, malformation, nerve

## Abstract

La vraie macrodactylie représente une anomalie congénitale rare d'étiologie inconnue, caractérisée par une augmentation de taille de tous les éléments d'un ou plusieurs rayons de la main. Elle est habituellement isolée et comporte une infiltration fibrograisseuse prédominant en palmaire. Elle peut être expliquée par des anomalies d'ordre embryologique ou neurogène; avec ou sans orientation nerveuse selon que le territoire concerné par la macrodactylie comporte une augmentation du volume d'un nerf majeur, le plus souvent le médian. Du point de vue évolutif, il faut distinguer la forme statique (présente à la naissance et restant stable avec la croissance) de la forme progressive (ou la croissance est disproportionnée). Certains syndromes, tumeurs ou malformations peuvent comporter une augmentation de volume d'un doigt sans qu'on puisse parler de vraie macrodactylie. Cette malformation engendre un handicap fonctionnel mais également un préjudice esthétique. Le traitement comporte non seulement la chirurgie mais aussi une prise en charge rééducative.

## Abstract (en)

Real macrodactylia is a rare congenital abnormality of unknown etiology, characterized by excessive growth of the anatomical structures of one or several rays of the hand. It is usually isolated and causes a fibrofatty infiltration mainly involving the palm. It can be caused by embryonic or neurogenic abnormalities with or without nerve involvement according to whether macrodactylia has led to an increase in the volume of a major nerve, more often the median. From an evolutionary point of view, it is necessary to distinguish between static macrodactylia (present at birth and remaining stable during growth) and progressive macrodactylia (with a disproportionate growths). Some other syndromes, tumors or abnormalities can cause an increase in finger volume. This abnormality results in functional disability but also in disfigurement. Treatment is based on surgery and rehabilitation.

## Introduction

La vraie macrodactylie de la main est une malformation rare, isolée, d'étiologie inconnue. Elle se caractérise par une augmentation de taille d'un ou plusieurs doigts, cette hypertrophie peut être statique ou progressive et ne doit pas être confondue avec une mégalodactylie syndromique et pseudo macrodactylie. Cette malformation est responsable d'un retentissement psychique et fonctionnel imposant une prise en charge chirurgicale précoce.

## Patient et observation

**Figure 1:**
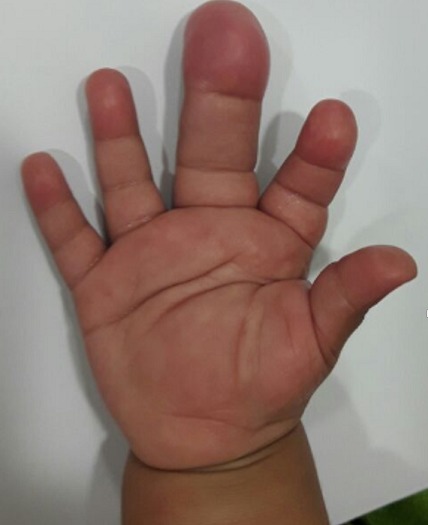
Macrodactylie du 3^ème^ doigt de la main droite: a noter la tuméfaction de la paume de la main

**Figure 2:**
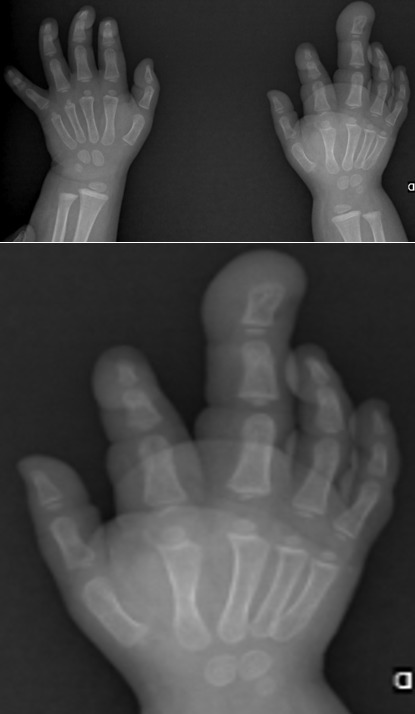
Radiographie standard radiographie des mains montrant une macrodactylie du 3^ème^ rayon de la main droite. Noter l'opacité des parties molles palmaires

**Figure 3:**
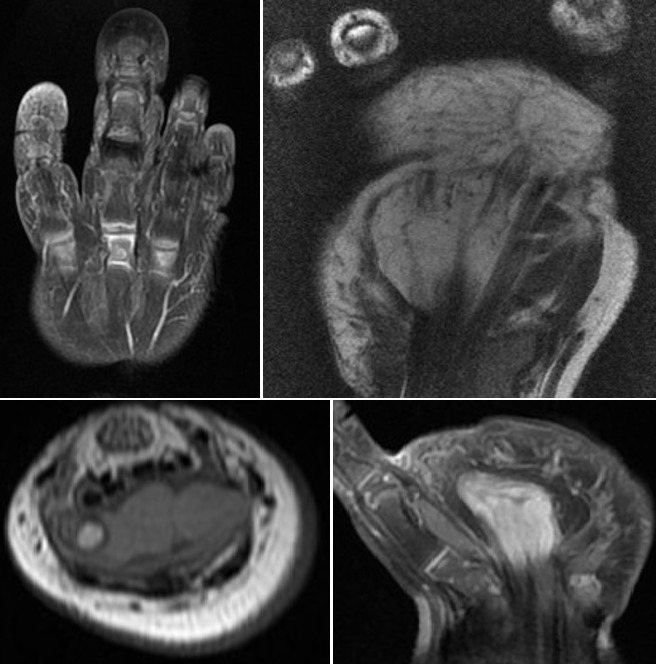
Echographie de la tuméfaction de la paume de la main droite

Il s'agit d'un enfant de sexe féminin, âgée de 1 an, sans antécédents pathologiques particuliers et à comportement psychomoteur normal. Elle a consulté pour une masse de la paume de la main et une hypertrophie du majeur de la main droite évoluant de façon progressive depuis la naissance et devenant de plus en plus douloureuse. Le bilan clinique a objectivé une augmentation globale de taille concernant aussi bien l'os que les tissus mous du troisième rayon sans caractère pulsatile ni compressible. La peau en regard était lisse. La mobilité des articulations métacarpo-phalangienne et inter-phalangiennes était conservée. Il s'y associait une masse molle, non douloureuse de la paume de la main (Figure 1). La radiographie standard a montré une hypertrophie osseuse harmonieuse et augmentation de taille en largeur, en longueur et en hauteur des 3 phalanges du 3^ème^ doigt de la main droite avec un élargissement de l'espace inter métacarpien de part et d'autre. Les interlignes articulaires intermétacarpo-phalangienne et interphalangiennes étaient respectées (Figure 2). L'échographie a retrouvé un élargissement du nerf médian avec prolifération graisseuse dissociant les fascicules nerveux. L'IRM a objectivé l'augmentation de volume des différents constituants du doigt y compris le nerf médian qui se présentait en hyposignal en T1 et T2 avec des fascicules dissociés par un infiltrat graisseux. Les os étaient hypertrophiés mais de signal normal (Figure 3).

## Discussion

### Généralités

La macrodactylie est définie par une augmentation de volume d'un ou de plusieurs doigts. En réalité, plusieurs états peuvent aboutir à l'augmentation de volume d'un doigt, comme la neurofibromatose, les lymphangiomes, les hémangiomes et fistules artérioveineuses, un muscle anormal, les syndromes d'Albright ou de Klippel-Trenaunay-Weber. La vraie macrodactylie est rare et doit être distinguée de ces autres causes. L'étiologie est inconnue. La théorie la plus communément admise est l'association entre une anomalie nerveuse et la macrodactylie. En effet, la forme la plus fréquente appelée lipofibromatose est associée à un élargissement du nerf digital innervant la région présentant une macrodactylie [1].

### Ethiopathogénie

La macrodactylie n'est pas une maladie héréditaire. Son étiologie n'est pas encore déterminée, deux théories expliquent sa pathogénie: la théorie de Barskey par anomalie localisée des récepteurs aux facteurs de croissance tissulaire. La théorie neurogène défendue par Inglis qui a souligné l'effet de l'hypertrophie nerveuse dans les formes dite « à orientation nerveuse » [2].

### Anatomopathologie

Il existe une prolifération du tissu graisseux avec des lobules larges et sombres. La peau est épaissie. Les tendons sont normaux ou élargis et la gaine de fléchisseurs épaissie expliquant parfois la présence de doigts à ressaut. La paroi des artères digitales est épaissie mais la lumière apparaît élargie. Les tissus sont peu vascularisés, expliquant les complications postopératoires à type de nécrose. Les nerfs digitaux et parfois les nerfs médian et ulnaire, dans les formes à orientation nerveuse, sont infiltrés par du tissu adipeux. L'endonèvre et le périnèvre sont fibreux. Les axones, bien que déformés, restent normaux en taille et en calibre. Du point de vue squelettique, les centres d'ossification enchondrale ne sont pas affectés. En revanche, une augmentation de l'activité fibroblastique est présente au niveau du périoste qui est épaissi. Dans ce tissu, on trouve plusieurs nodules composés de cartilage hyalin avec parfois formation de cartilage et d'os. Il existe une accélération du remodelage comme en témoigne la présence de nombreux ostéoblastes et ostéoclastes [3].

### Clinique

Du point de vue clinique, l'atteinte est unilatérale, dans 95% des cas, sans atteinte prédominante du côté droit ou gauche. Une légère prédominance masculine est observée. Les rayons digitaux les plus atteints sont par ordre décroissant l'index, le médius, le pouce, l'annulaire, puis l'auriculaire. L'atteinte multidigitale est plus fréquente (70%) que l'atteinte unidigitale: les associations pouce–index et index–médius sont les plus fréquentes. Le doigt apparaît hypertrophique de façon globale en longueur et en largeur. En cas d'atteinte d'un rayon radial, la déformation est en inclinaison ulnaire. À l'opposé, l'auriculaire présente une inclinaison radiale. Lorsque deux doigts adjacents sont atteints, ils présentent une courbure inversée de telle sorte que les pulpes sont en contact. On retrouve également une hyperextension des articulations interphalangiennes. Pour le pouce, la déformation est en abduction et en hyperextension. Au niveau digital, l'hypertrophie est plus marquée au niveau des phalanges distales. La croissance osseuse est accélérée avec un avancement de l'âge osseux. Cette croissance osseuse s'estompe lors de la fermeture des cartilages de conjugaison. Les articulations s'enraidissent progressivement avec l'âge, soit par rétraction capsulaire, soit du fait de la non-utilisation, soit par ankylose osseuse ou par dépôts cartilagineux périarticulaires (forme hyperostéotique). Les nerfs sont épaissis avec des trajets sinueux. Une infiltration graisseuse est constante, sans plan de clivage avec le tissu neural et avec parfois une extension proximale aux nerfs médian et cubital. Il faut noter la fréquence du syndrome du canal carpien chez les adultes porteurs de macrodactylie. La sensibilité est le plus souvent normale. Le retentissement psychologique n'est pas négligeable, incitant l'enfant à cacher sa main [4].

### Moyens d'imagerie

**La radiographie standard:** Est l'examen demandé en première intention. Les radiographies montrent, dans les formes sévères, outre une augmentation de volume des phalanges, une hypertrophie des métacarpiens ainsi qu'un élargissement de l'espace inter-métacarpien lié à une hypertrophie d'un muscle interosseux. À l'âge adulte peuvent apparaître des ostéophytes, des masses ostéocartilagineuses autour des articulations et des signes d'arthrose [2-4].

**L'échographie:** Peut retrouver un élargissement fusiforme du nerf avec prolifération graisseuse dissociant les fascicules nerveux et un éventuel rétrécissement dans le canal carpien [3, 4].

**IRM:** l'IRM confirme l'augmentation de volume du nerf avec un aspect sinueux et apprécie l'extension proximale de l'infiltration graisseuse [4]. Cet épaississement nerveux est en hypersignal en T1 et T2 [5].

**L'artériographie:** Si elle était pratiquée ne montre aucune malformation artério-veineuse [6].

### Formes cliniques

**La forme statique:** Dans laquelle la macrodactylie est présente dès la naissance, le ou les doigts atteints grandissent parallèlement à la croissance des autres doigts.

**La forme progressive ou dynamique:** Dans laquelle la macrodactylie n'est pas présente à la naissance, le ou les doigts atteints croissent de manière accélérée, devenant de plus en plus grands et atteignant vite la taille d'un doigt adulte.

**Les formes symétriques et asymétriques:** Holmes distinguait dans les symétriques, tous les éléments du doigt sont hypertrophies alors que dans les asymétriques, l'hypertrophie osseuse et graisseuse est prédominante [7].

### Méthodes thérapeutiques

Les traitements sont à adapter au cas par cas, en fonction du volume du ou des rayons atteints, du mode évolutif et de l'âge du patient. Ils sont difficiles et souvent décevants nécessitant plusieurs temps opératoires et comportent: des interventions visant à ralentir la croissance chez l'enfant, essentiellement par des épiphysiodèses, des gestes de remodelage des parties molles plus ou moins combinés à des raccourcissements osseux distaux, des ostéotomies de réaxation et un stripping des nerfs collatéraux, des amputations, proposées dans les formes progressives sévères [8].

## Conclusion

La macrodactylie est une maladie congénitale dans laquelle le tissu mou, l'os et la graisse des doigts affectés sont hypertrophiés. C'est une pathologie rare, d'étiologie inconnue. Les deux hypothèses essayant d'expliquer sa pathogénie sont la théorie embryologique ou neurogène. Un examen clinique détaillé est indispensable afin de différencier les pseudo-macrodactylies syndromiques des vraies macrodactylies. Les moyens d'imagerie en coupes permettent d'affirmé les forme à orientation neurogène devant un nerf épaissi, sinueux et infiltré par la graisse. Le traitement est à adapter au cas par cas pour améliorer le pronostic globalement mauvais des macrodactylies.

## Conflits d'intérêts

Les auteurs ne déclarent aucun conflit d'intérêts.
